# Supplementary material for: Three-dimensional CRISPR screening reveals epigenetic interaction with anti-angiogenic therapy
Source: Commun Biol. 2021 Jul 15;4:878. doi: 10.1038/s42003-021-02397-3 (PMC8282794; doi:10.1038/s42003-021-02397-3)
Supplement: Supplementary file 2 — Supplementary Information [file 42003_2021_2397_MOESM2_ESM.pdf]

Three-dimensional CRISPR screening reveals epigenetic interaction with anti-angiogenic therapy

Michael Y. He[1,2], Michael M. Halford[1], Ruofei Liu[1,2], James P. Roy[1,2], Zoe L. Grant[3,4], Leigh Coultas[3,4], Niko Thio[5], Omer Gilan[2,6], Yih-Chih Chan[6], Mark A. Dawson[2,6,7,8], Marc G. Achen[1,2,9] & Steven A. Stacker[1,2,9*]

[1]Tumour Angiogenesis and Microenvironment Program, Peter MacCallum Cancer Centre, 305 Grattan St, Melbourne, Victoria, Australia, 3000.

[2]Sir Peter MacCallum Department of Oncology, The University of Melbourne, Parkville, Victoria, Australia, 3010.

[3]Epigenetics and Development Division, The Walter and Eliza Hall Institute of Medical Research, Parkville, Victoria, Australia, 3052.

[4]Department of Medical Biology, The University of Melbourne, Parkville, Victoria, Australia, 3010.

[5]Bioinformatics Core, Peter MacCallum Cancer Centre, 305 Grattan St, Melbourne, Victoria, Australia, 3000.

[6]Translational Haematology Program, Peter MacCallum Cancer Centre, 305 Grattan St, Melbourne, Victoria, Australia, 3000.

[7]Centre for Cancer Research, The University of Melbourne, Parkville, Victoria, Australia, 3010.

[8]Department of Haematology, Peter MacCallum Cancer Centre, 305 Grattan St, Melbourne, Victoria, Australia, 3000.

[9]Department of Surgery, Royal Melbourne Hospital, The University of Melbourne, Parkville, Victoria, Australia, 3050.

Corresponding author: Steven Stacker, Tumour Angiogenesis and Microenvironment Program, Peter MacCallum Cancer Centre, 305 Grattan St, Melbourne, VIC 3000, Australia; Phone +61 3 8559 7106; Email: Steven.Stacker@petermac.org.

Supplementary Information

Supplementary Methods

Individual sgRNA design and cloning

For cloning individual sgRNAs into the lentiCRISPRv2 backbone (Addgene, #52961; Supplementary Fig. 3a), a single-stranded oligonucleotide (oligo; displayed below) was synthesized by IDT as an Ultramer product. This encoded a 20 nt sgRNA sequence plus a partial sequence of the human U6 promoter (underlined) and a partial sequence of the chRNA scaffold (double underlined):

5'-ATCTTGTGGAAAGGACGAAACACC$(N)_{20}$GTTTTAGAGCTAGAAATAGCAAGTT-3'

This design allowed for single-step cloning of the sequence encoding the sgRNA into the lentiCRISPRv2 backbone. Cloning was performed using *Bsm*BI (NEB, #R0580S) digested lentiCRISPRv2 plasmid, the oligo design shown above and the NEBuilder HiFi DNA Assembly Master Mix according to the manufacturer's instructions. The plasmid encoding an sgRNA targeting the gene *XXX* is referred to as lentiCRISPRv2_sgXXX. The purified LV transfer plasmid lentiCRISPRv2_sgXXX was used for LV production. For evaluation purposes, an oligo encoding an sgRNA targeting the *AAVS1* locus (sequence: 5'-GGGGCCACTAGGGACAGGAT-3') was cloned into lentiCRISPRv2 as described above. The resulting LV transfer plasmid lentiCRISPRv2_sgAAVS1 was used for LV production.

Evaluation of CRISPR–Cas9 activity in ECs

ECs were transduced with the LV encoding lentiCRISPRv2_sgAAVS1 at an MOI of 0.3–0.4. Puromycin treatment at 0.5 µg/mL was started 48 h post-transduction (referred to hereafter as days post-infection (dpi) 2) and applied for three days (Supplementary Fig. 3b). On dpi 5, puromycin concentration was reduced to, and maintained at, 0.3 µg/mL. Cells were harvested for total protein extraction or for gDNA extraction using QuickExtract DNA Extraction Solution on dpi 5, 6, 7, 10 and 12 (Supplementary Fig. 3b). Total protein and gDNA from untransduced cells were also extracted and used as a negative control for assessing Cas9 protein expression and gene editing efficiency, respectively.

Cas9 protein expression was assessed by western blotting. For evaluation of CRISPR–Cas9-induced genomic cleavage, part of the *AAVS1* locus was amplified by PCR (using Q5 Hot Start High-Fidelity 2× Master Mix, forward primer AAVS1_R1: 5'-CCCCGTTCTCCTGTGGATTC-3' and reverse primer AAVS1_R1: 5'-ATCCTCTCTGGCTCCATCGT-3'). The PCR product was subjected to Sanger sequencing. The sequencing results were decomposed and analyzed using a bioinformatics tool, Tracking of Indels by DEcomposition (TIDE)[1].

Quantitative reverse transcription PCR (qRT-PCR)

Complementary DNA (cDNA) was prepared from total RNA by reverse transcription (High Capacity cDNA Reverse Transcription Kit, Life Technologies) according to the manufacturer's instructions. Quantitative PCR was performed on a StepOnePlus Real-Time PCR System (Life Technologies) using cDNA, TaqMan Gene Expression Assays (BRD2 Hs01121986_g1, BRD3 Hs00201284_m1, BRD4 Hs04188087_m1, GAPDH Hs99999905_m1; Life Technologies) and TaqMan Fast Advanced Master Mix (Life Technologies) according to the manufacturer's instructions. The relative expression levels of target genes were calculated by normalization to that of the reference gene *GAPDH* using the $2^{-\Delta\Delta CT}$ method[2].

Supplementary Figure 1

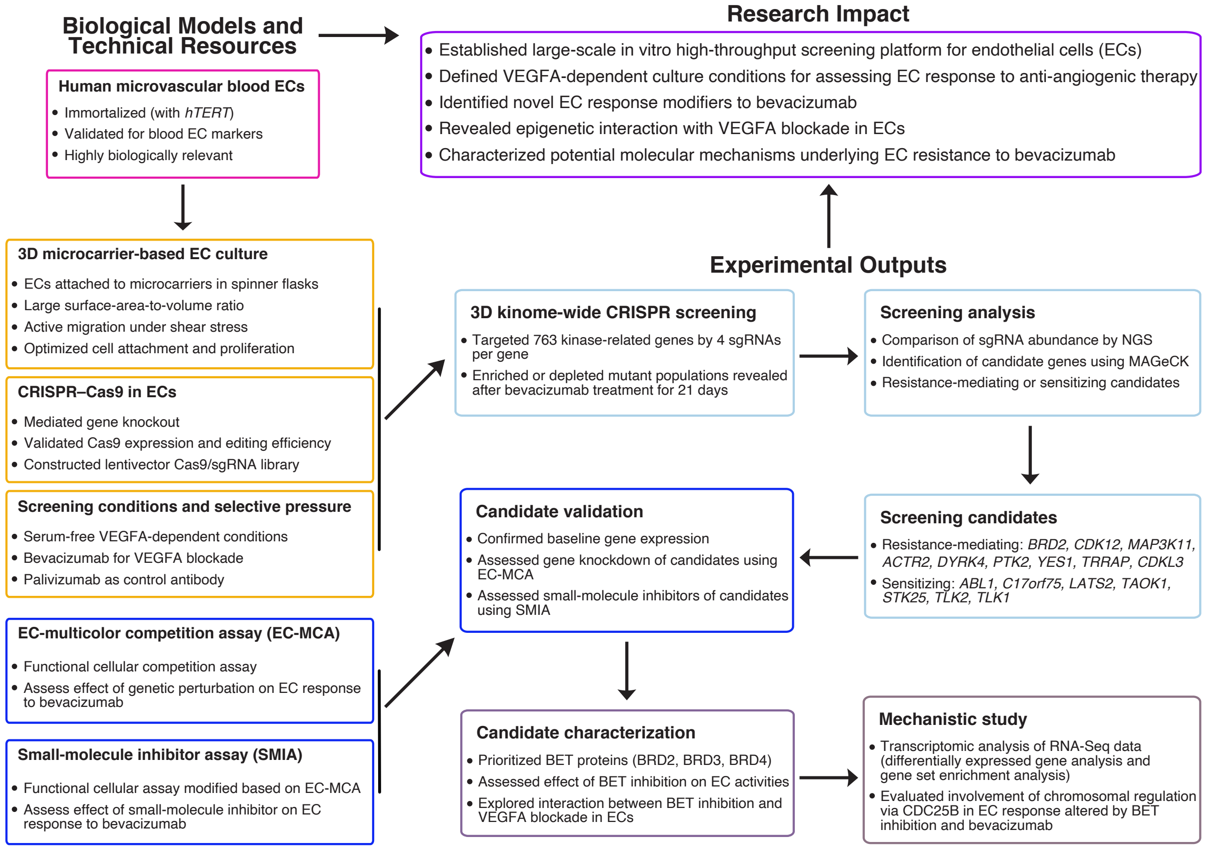

Supplementary Figure 1. Flow chart of the study. In this study, we aim to identify the molecular modifiers of human microvascular blood endothelial cell (EC) response to anti-angiogenic therapy. To this end, we have established three-dimensional (3D) culture system to allow large-scale in vitro EC culture to meet the high demand for cell number in a CRISPR screen. We have also developed effective CRISPR–Cas9 gene editing in ECs and VEGFA-dependent culture conditions for assessing EC response to the VEGFA neutralizing antibody bevacizumab. We performed the 3D CRISPR screen by combining these three key elements and identified novel EC response modifiers to bevacizumab. Further candidate validation and characterization experiments confirmed an interaction between bromodomain and extraterminal domain (BET) inhibition and VEGFA blockade which was associated with altered EC response. Mechanistically, this may be related to chromosomal regulation via CDC25B. Our study not only provides technical advances to enable large-scale pooled in vitro screening in ECs but also reveals molecular insight into regulation of EC response to anti-angiogenic therapy. hTERT, human telomerase reverse transcriptase; MAGeCK, Model-based Analysis of Genome-wide CRISPR/Cas9 Knockout; NGS, next-generation sequencing.

Supplementary Figure 2

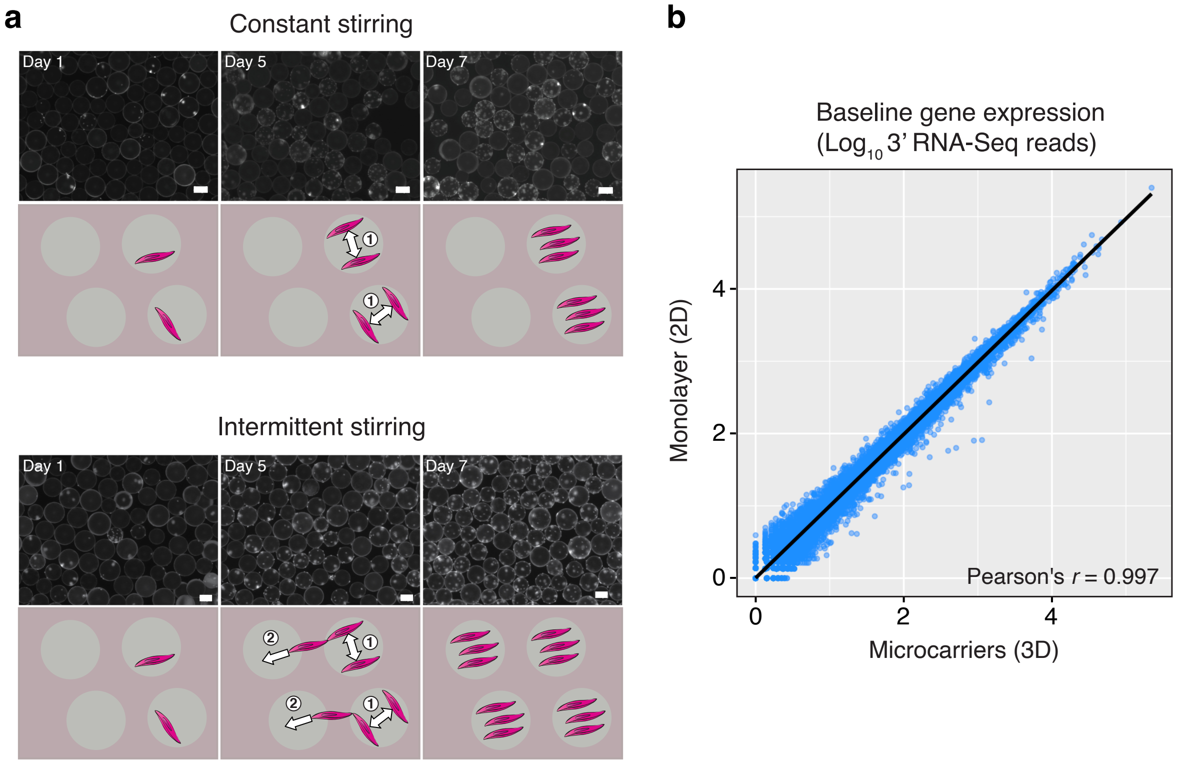

Supplementary Figure 2. EC migration via bead-to-bead transfer in microcarrier-based culture and baseline gene expression comparison between monolayer and microcarrier-based culture. a Cell distribution on microcarriers with constant stirring or intermittent stirring (11 h on/1 h off) is shown schematically below images of Hoechst 33342-stained nuclei. Cells occupied the microcarriers by proliferation on the same microcarrier (mechanism 1) and/or migration between adjacent microcarriers (mechanism 2) during the incubation period. Scale bars, 100 µm. **b** Correlation of baseline gene expression based on data from RNA-Seq performed in ECs cultivated using monolayer or microcarrier-based cell culture. Pearson correlation coefficient (Pearson's *r*) was calculated using Microsoft Excel.

Supplementary Figure 3

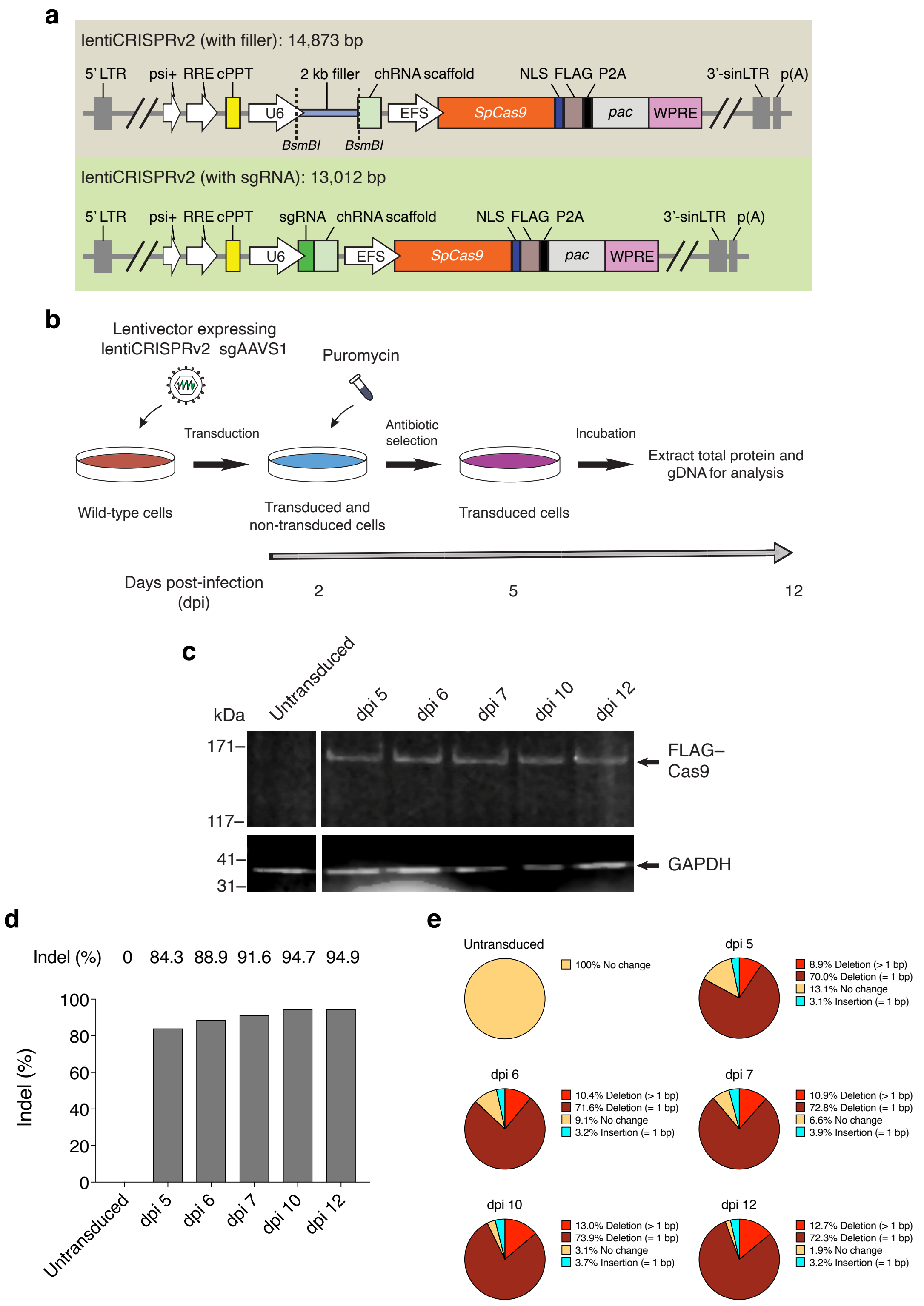

Supplementary Figure 3. Evaluation of genomic cleavage induced by CRISPR–Cas9 in ECs. a Important features of the lentivector transfer plasmid lentiCRISPRv2. **b** Timeline of sample preparation for evaluating CRISPR–Cas9 activity. **c** Western blotting analysis of FLAG–Cas9 protein expression. **d** Total indel frequency as analyzed from decomposition of Sanger sequencing results using TIDE. Percentages represent results from one experiment. **e** Characterization of indels induced by CRISPR–Cas9 from the analysis in (**d**).

Supplementary Figure 4

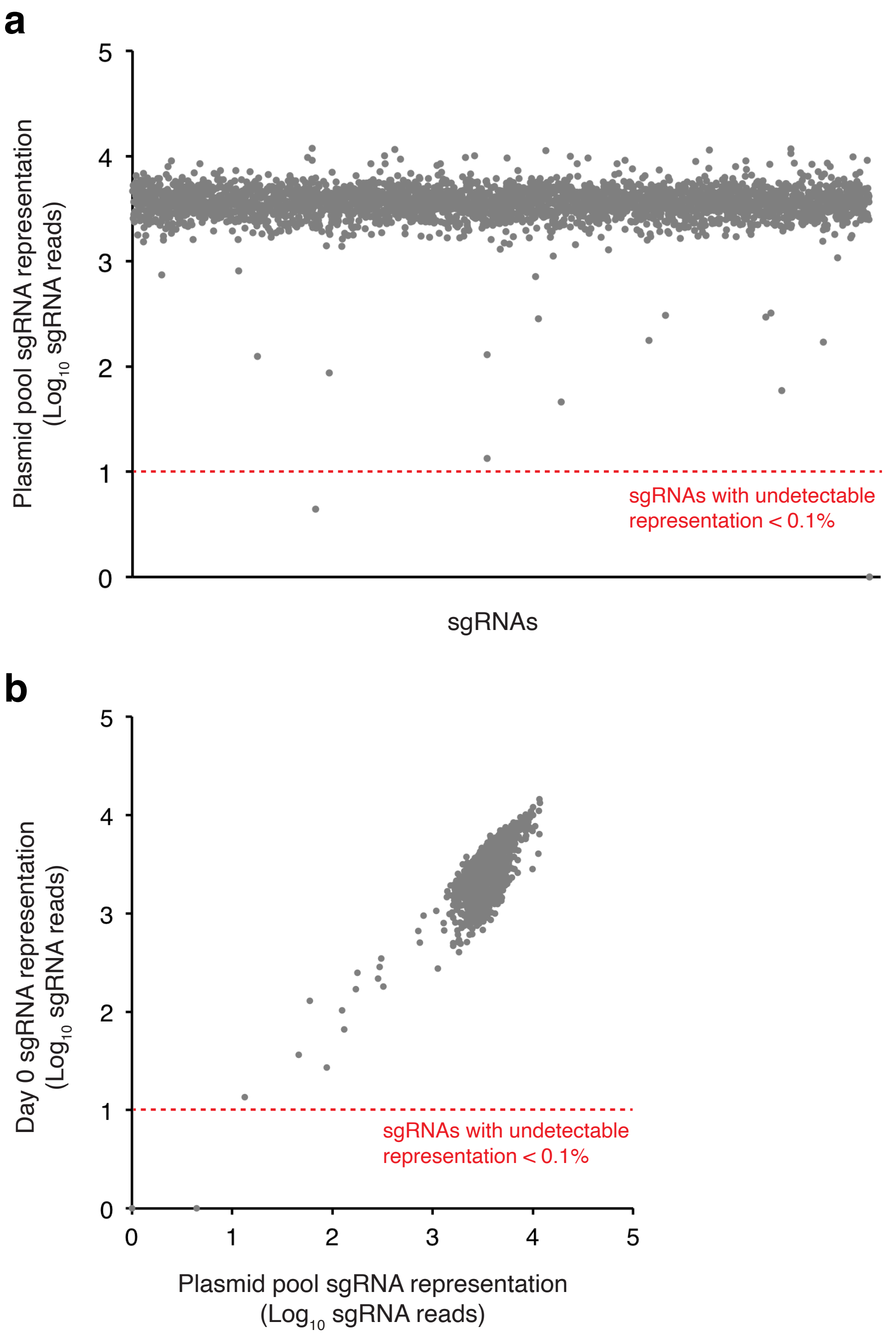

Supplementary Figure 4. Deep sequencing analysis of sgRNA representation. a Evaluation using the plasmid pool. **b** Comparison between the plasmid pool and microcarrier culture samples collected on day 0. Individual sgRNA representation is displayed by the normalized $\log_{10}$ sgRNA reads generated by deep sequencing using the plasmid pool of the Cas9/sgRNA library or gDNA isolated from transduced cells on day 0 (i.e. the beginning of screening selection). sgRNAs which have fewer than 10 reads (the red dotted line) are defined as under-represented.

Supplementary Figure 5

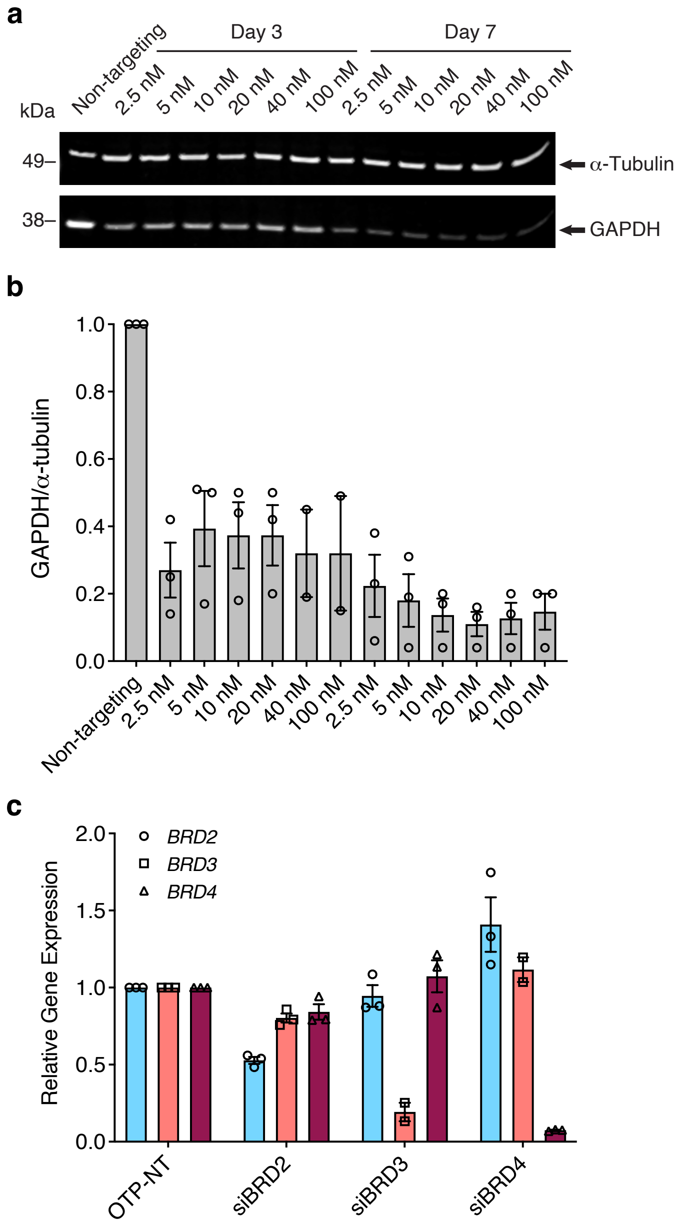

Supplementary Figure 5. Evaluation of siRNA-mediated knockdown in ECs. a Western blotting analysis of siRNA-mediated knockdown of GAPDH. α-tubulin was used as a loading control. **b** Quantification of GAPDH protein expression. GAPDH signal is normalized to that of α-tubulin. The normalized GAPDH signals in all samples are expressed relative to the non-targeting control. Error bars represent ± SEM (*n* = 3 independent experiments). ECs were transfected with increasing concentrations of siGAPDH or the non-targeting siRNA pool (OTP-NT; final concentration in medium, 10 nM) for 24 h. Cells were then maintained in EGM-2MV. Cell lysates were prepared three days or seven days after the beginning of transfection, i.e., on day 3 or day 7, respectively (for cells transfected with OTP-NT, the lysates were prepared on day 7). **c** qRT-PCR analysis of siRNA-mediated knockdown of *BRD2*, *BRD3* or *BRD4*. Gene expression is normalized to that of *GAPDH* in each sample. The normalized gene expression in all samples is expressed relative to the non-targeting control (OTP-NT). Error bars represent ± SEM (*n* = 3 independent experiments). ECs were transfected for 24 h and subsequently maintained in EGM-2MV. Total RNA was isolated five days after the beginning of transfection.

Supplementary Figure 6

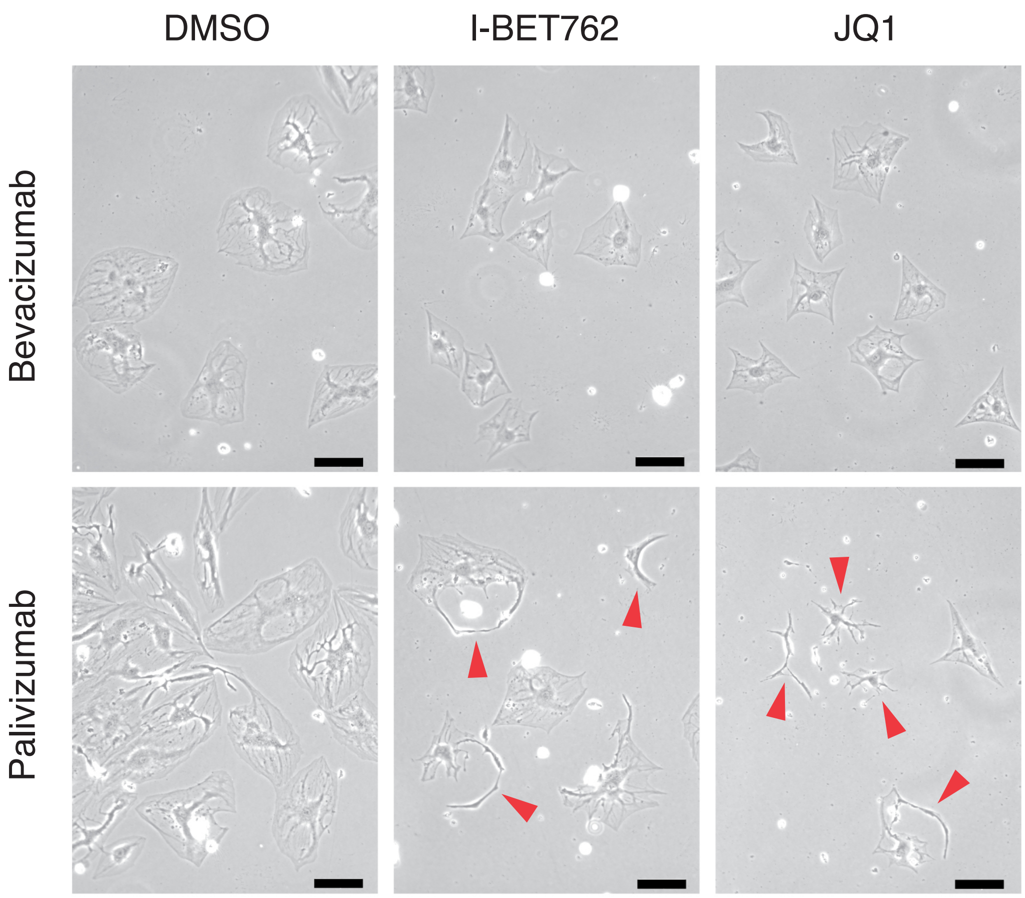

Supplementary Figure 6. Cell morphology during treatment with BETi in ESFM. ECs were treated with DMSO (0.1% v/v), I-BET762 (1000 nM) or JQ1 (300 nM) in ESFM plus bevacizumab or palivizumab (cells spread themselves when cultured in serum-free conditions regardless of treatment). Phase-contrast images were taken on day 9 of BETi treatment in ESFM. Red arrowheads indicate examples of cells with abnormal morphology when treated with BETi compared to DMSO. Scale bars, 100 µm.

Supplementary Figure 7

Uncropped blots for Supplementary Figure 3c

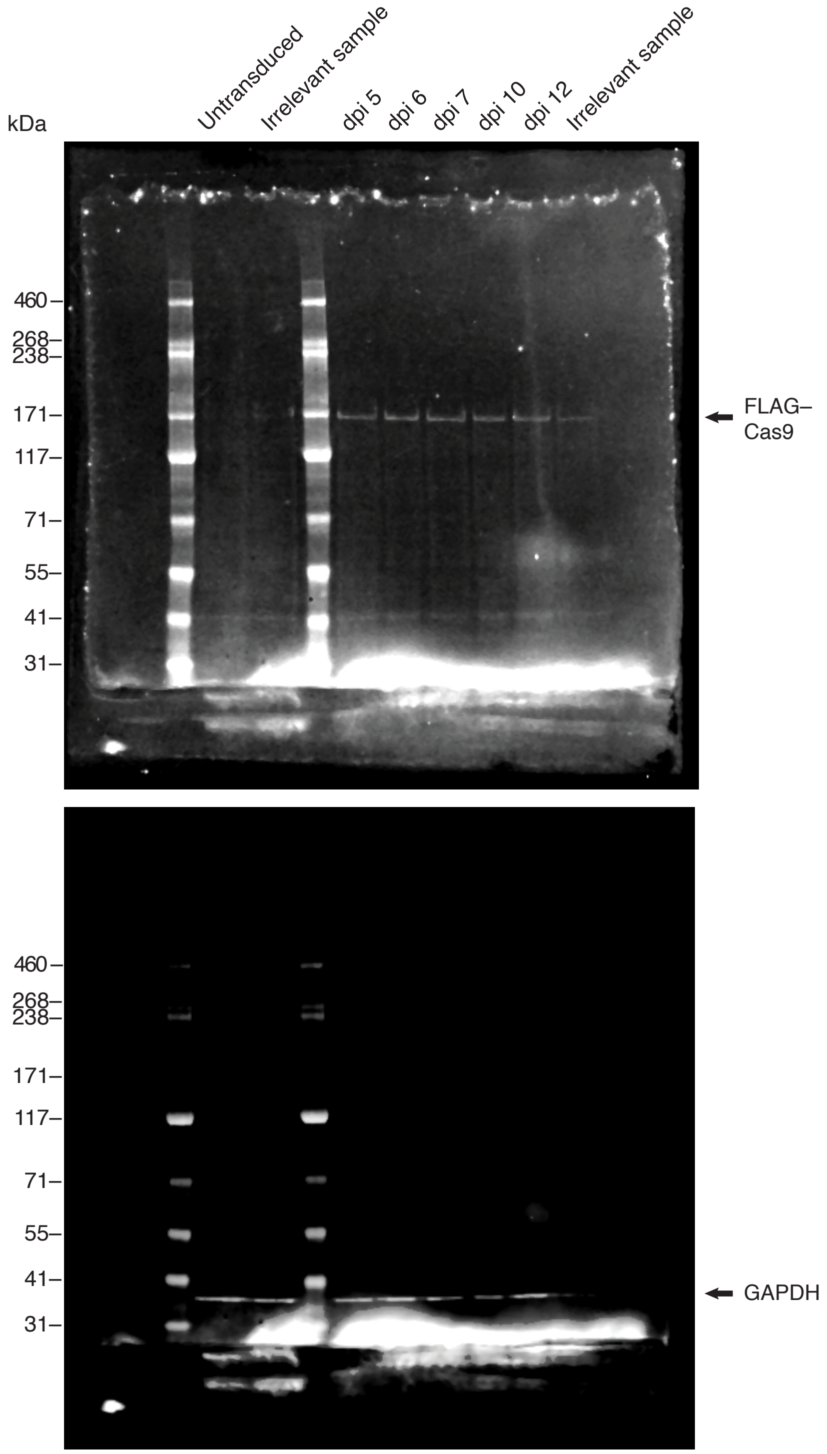

Supplementary Figure 7. Uncropped blots for Supplementary Figure 3c.

Supplementary Figure 8

Uncropped blot for Supplementary Figure 5a

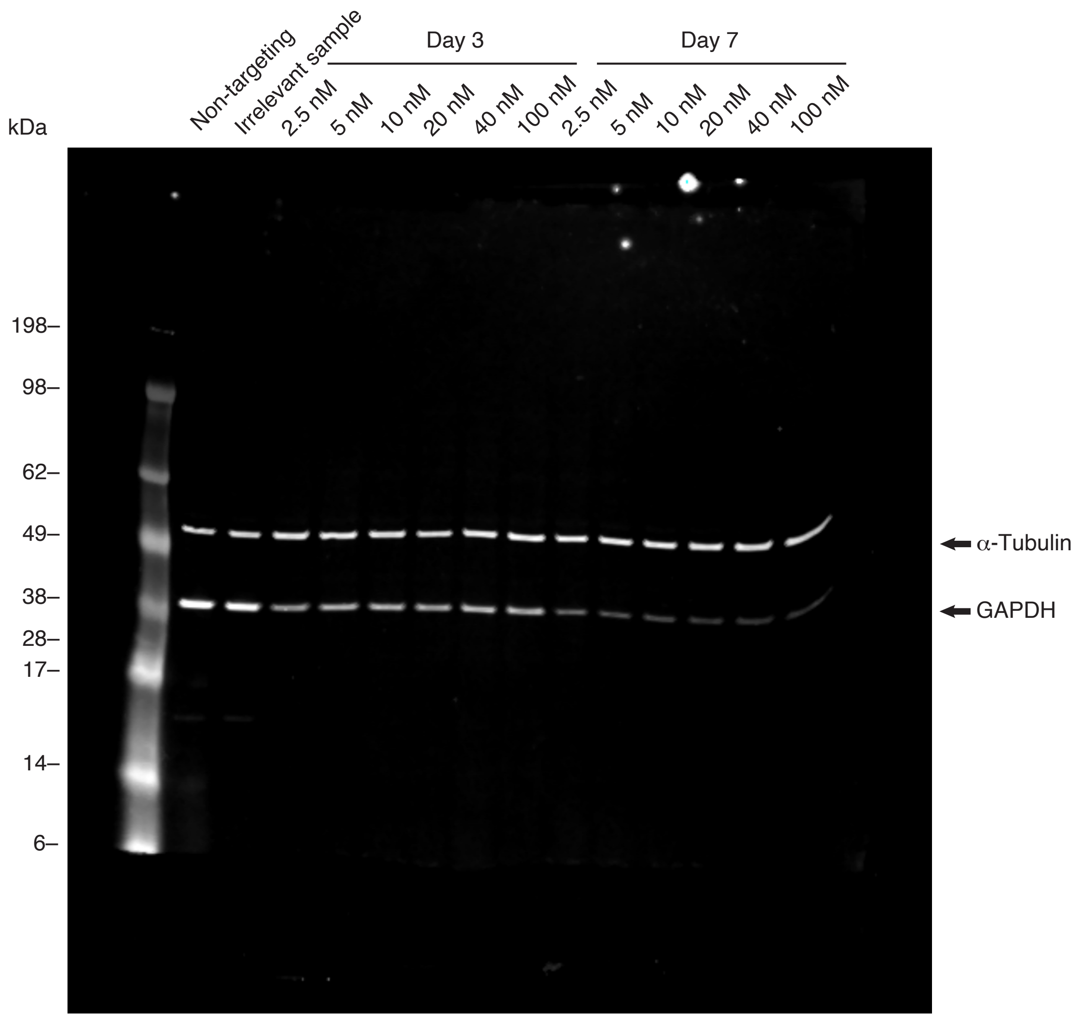

Supplementary Figure 8. Uncropped blot for Supplementary Figure 5a.

Supplementary Table 1. Composition of BCM-2+ and MCA wash solution.

**BCM-2+**	
**Component**	**Final concentration**
EBM-2 (Lonza, #CC-3156)	n/a
1 M HEPES (4-(2-hydroxyethyl)-1-piperazineethanesulfonic acid; Life Technologies, #15630080)	10 mM
FBS (Lonza, #CC-3202)	1% v/v
75 mM ascorbic acid (Sigma, #A4034)/500 mM 2-phosphoascorbic acid (Sigma, #49752) in Milli-Q water neutralized to pH 7.4 at RT	75 µM/500 µM
50 mg/mL gentamicin (Life Technologies, #15750060)	10 µg/mL
2.5 mg/mL hydrocortisone (Sigma, #H0888) in 100% ethanol	1 µg/mL

NOTE: 0.3 or 0.5 *µ*g/mL puromycin-2HCl (Life Technologies, #A1113803) was added when the puromycin resistance gene was expressed.

**MCA wash solution**	
**Component**	**Final concentration**
EBM-2 (Lonza, #CC-3156)	n/a
1 M HEPES (4-(2-hydroxyethyl)-1-piperazineethanesulfonic acid; Life Technologies, #15630080)	10 mM
20% w/v AlbuMAX II (Life Technologies, #11021037) in 1× DPBS	0.25% v/v
75 mM ascorbic acid (Sigma, #A4034)/500 mM 2-phosphoascorbic acid (Sigma, #49752) in Milli-Q water neutralized to pH 7.4 at RT	75 µM/500 µM
50 mg/mL gentamicin (Life Technologies, #15750060)	10 µg/mL

Supplementary Table 2. Composition of endothelial serum-free medium for microcarrier-based culture of endothelial cells (ESFM-3D) plus bevacizumab or palivizumab.

**ESFM-3D plus bevacizumab or palivizumab**	
**Component**	**Final concentration**
EBM-2 (Lonza, #CC-3156)	n/a
1 M HEPES (4-(2-hydroxyethyl)-1-piperazineethanesulfonic acid; Life Technologies, #15630080)	20 mM
20% w/v AlbuMAX II (Invitrogen, #11021037) in 1× DPBS	0.25% v/v
50 × Nutridoma-CS (Roche, Sydney, NSW, Australia, #11363743001)	0.75× (1.5 % v/v)
Chemically defined lipid concentrate (Life Technologies, #11905031)	0.50% v/v
Monoclonal antibody (bevacizumab or palivizumab)	100 nM
100 × MEM Non-Essential Amino Acids solution (Invitrogen, #11140050; neutralized to pH 7.4 at RT)	1×
2.5 mg/mL hydrocortisone (Sigma, #H0888) in 100% ethanol	1 µg/mL
200 mM dibutyryl cyclic adenosine monophosphate (Sigma, #D0627) in 1× DPBS	0.5 mM
60 mM serotonin-HCl (5-hydroxytryptamine-HCl; Sigma, #H9523) in 1× DPBS	700 µM
2 mM sphingosine-1-phosphate (Avanti Polar Lipid, Inc., Alabaster, AL, USA, #860492P) in 95% v/v methanol	200 nM
1 mg/mL heparin (Sigma, #H3149) in Milli-Q water	0.1 µg/mL
75 mM ascorbic acid (Sigma, #A4034)/500 mM 2-phosphoascorbic acid (Sigma, #49752) in Milli-Q water neutralized to pH 7.4 at RT	75 µM/500 µM
1000 × Trace Elements C (Mediatech, Inc., Manassas, VA, USA, #25-023-CI)	1×
50 mg/mL gentamicin (Invitrogen, #15750060)	1 µg/mL
50 µg/mL rhFGF-2 (PeproTech, Rocky Hill, NJ, USA, #100-18B)	10 ng/mL
100 *µ*g/mL Long R3 rhIGF1 (PeproTech, #100-11R3)	27.5 ng/mL
100 µg/mL $rhVEGFA_{165}$ (PeproTech, #100-20)	20 ng/mL (500 pM homodimer)
10 × LIM1863-*Mph* serum-free conditioned medium	0.1× (1% v/v)
1 mg/mL puromycin-2HCl (Life Technologies, #A1113803)	0.3 µg/mL

Supplementary Table 3. Composition of endothelial serum-free medium for monolayer culture of endothelial cells (ESFM-2D) plus bevacizumab or palivizumab.

**ESFM-2D plus bevacizumab or palivizumab**	
**Component**	**Final concentration**
EBM-2 (Lonza, #CC-3156)	n/a
1 M HEPES (4-(2-hydroxyethyl)-1-piperazineethanesulfonic acid; Life Technologies, #15630080)	20 mM
20% w/v AlbuMAX II (Life Technologies, #11021037) in 1× DPBS	0.25% v/v
50 × Nutridoma-CS (Roche, Sydney, NSW, Australia, #11363743001)	0.5× (1% v/v)
Chemically defined lipid concentrate (Life Technologies, #11905031)	0.50% v/v
Monoclonal antibody (bevacizumab or palivizumab)	50 nM
100 × MEM Non-Essential Amino Acids solution (Invitrogen, #11140050; neutralized to pH 7.4 at RT)	1×
2.5 mg/mL hydrocortisone (Sigma, #H0888) in 100% ethanol	1 µg/mL
200 mM dibutyryl cyclic adenosine monophosphate (Sigma, #D0627) in 1× DPBS	0.5 mM
60 mM serotonin-HCl (5-hydroxytryptamine-HCl; Sigma, #H9523) in 1× DPBS	300 µM
2 mM sphingosine-1-phosphate (Avanti Polar Lipid, Inc., Alabaster, AL, USA, #860492P) in 95% v/v methanol	200 nM
1 mg/mL heparin (Sigma, #H3149) in Mill-Q water	0.1 µg/mL
75 mM ascorbic acid (Sigma, #A4034)/500 mM 2-phosphoascorbic acid (Sigma, #49752) in Mill-Q water neutralized to pH 7.4 at RT	75 µM/500 µM
1000 × Trace Elements C (Mediatech, Inc., Manassas, VA, USA, #25-023-CI)	1×
50 mg/mL gentamicin (Life Technologies, #15750060)	1 µg/mL
500 ng/mL rhFGF-2 (PeproTech, Rocky Hill, NJ, USA, #100-18B)	82.5 pg/mL
50 µg/mL rhVEGFA$_{165}$ (PeproTech, #100-20)	10 ng/mL (250 pM homodimer)
10 × LIM1863-*Mph* serum-free conditioned medium	0.1× (1% v/v)

NOTE: 0.3 µg/mL puromycin-2HCl (Life Technologies, #A1113803) was added when the puromycin resistance gene was expressed.

Supplementary Table 4. Composition of endothelial serum-reduced medium (ESRM) plus bevacizumab or palivizumab.

**ESRM plus bevacizumab or palivizumab**	
**Component**	**Final concentration**
EBM-2 (Lonza, #CC-3156)	n/a
1 M HEPES (Life Technologies, #15630080)	10 mM
Human serum (Sigma, #6914)	1% v/v
75 mM ascorbic acid (Sigma, #A4034)/500 mM 2-phosphoascorbic acid (Sigma, #49752) in Milli-Q water neutralized to pH 7.4 at RT	75 µM/500 µM
50 mg/mL gentamicin (Life Technologies, #15750060)	10 µg/mL
2.5 mg/mL hydrocortisone (Sigma, #H0888) in 100% ethanol	1 µg/mL
Monoclonal antibody (bevacizumab or palivizumab)	50 nM
50 µg/mL rhFGF-2 (PeproTech, Rocky Hill, NJ, USA, #100-18B)	1.2 ng/mL
100 *µ*g/mL Long R3 rhIGF1 (PeproTech, #100-11R3)	1.5 ng/mL
50 *µ*g/mL rhEGF (PeproTech, #AF-100-15)	3 ng/mL
50 µg/mL rhVEGFA$_{165}$ (PeproTech, #100-20)	10 ng/mL (250 pM homodimer)

NOTE: 0.3 *µ*g/mL puromycin-2HCl (Life Technologies, #A1113803) was added when the puromycin resistance gene was expressed.

Supplementary Table 5. Primers for preparing sequencing libraries for determining sgRNA representation.

**P5 XPR/LKO1 primer mix**	
**Name**	**Sequence (5'–3')**
P5_XPR/LKO1_1	AATGATACGGCGACCACCGAGATCTACACTCTTTCCCTACACGACGCTCTTCCGATCTTTGTGGAAAGGACGAAACACCG
P5_XPR/LKO1_2	AATGATACGGCGACCACCGAGATCTACACTCTTTCCCTACACGACGCTCTTCCGATCTCTTGTGGAAAGGACGAAACACCG
P5_XPR/LKO1_3	AATGATACGGCGACCACCGAGATCTACACTCTTTCCCTACACGACGCTCTTCCGATCTGCTTGTGGAAAGGACGAAACACCG
P5_XPR/LKO1_4	AATGATACGGCGACCACCGAGATCTACACTCTTTCCCTACACGACGCTCTTCCGATCTAGCTTGTGGAAAGGACGAAACACCG
P5_XPR/LKO1_5	AATGATACGGCGACCACCGAGATCTACACTCTTTCCCTACACGACGCTCTTCCGATCTCAACTTGTGGAAAGGACGAAACACCG
P5_XPR/LKO1_6	AATGATACGGCGACCACCGAGATCTACACTCTTTCCCTACACGACGCTCTTCCGATCTTGCACCTTGTGGAAAGGACGAAACACCG
P5_XPR/LKO1_7	AATGATACGGCGACCACCGAGATCTACACTCTTTCCCTACACGACGCTCTTCCGATCTACGCAACTTGTGGAAAGGACGAAACACCG
P5_XPR/LKO1_8	AATGATACGGCGACCACCGAGATCTACACTCTTTCCCTACACGACGCTCTTCCGATCTGAAGACCCTTGTGGAAAGGACGAAACACCG
**P7 primer**	
**Backbone sequence P7 XPR023 (5'–3')**	CAAGCAGAAGACGGCATACGAGATNNNNNNNNGTGACTGGAGTTCAGACGTGTGCTCTTCCGATCTCCAATTCCCACTCCTTTCAAGACCT
**Barcode ID**	**Barcode sequence (NNNNNNNN, 5'–3')**
A01	CGGTTCAA
A02	GCTGGATT
A03	TAACTCGG
A04	TAACAGTT
A05	ATACTCAA
A06	GCTGAGAA
A07	ATTGGAGG
A08	TAGTCTAA
A09	CGGTGACC

Supplementary References

1. Brinkman EK, Chen T, Amendola M, van Steensel B. Easy quantitative assessment of genome editing by sequence trace decomposition. *Nucleic Acids Res* **42**, e168 (2014).

2. Livak KJ, Schmittgen TD. Analysis of relative gene expression data using real-time quantitative PCR and the 2(-Delta Delta C(T)) Method. *Methods* **25**, 402-408 (2001).
