# Supplementary material for: Three-dimensional CRISPR screening reveals epigenetic interaction with anti-angiogenic therapy
Source: Commun Biol. 2021 Jul 15;4:878. doi: 10.1038/s42003-021-02397-3 (PMC8282794; doi:10.1038/s42003-021-02397-3)
Supplement: Supplementary file 3 — Description of Additional Supplementary File [file 42003_2021_2397_MOESM3_ESM.pdf]

Description of Additional Supplementary Files

File name: Supplementary Data 1
Description: Normalized sequencing reads from RNA-Seq analysis of baseline gene expression in human microvascular blood endothelial cells cultivated in microcarrier-based or monolayer culture (data from two independent experiments). Source data for Fig. 3a and Supplementary Figure 2b.

File name: Supplementary Data 2
Description: Detailed information about the 18 candidate genes identified from the 3D kinome-wide CRISPR screen.

File name: Supplementary Data 3
Description: MAGeCK analysis of the 3D kinome-wide CRISPR screen (data from two independent experiments), showing the complete sgRNA and gene ranking lists at day 12 and day 21 between bevacizumab (B) treatment arm and palivizumab (P) control arm. Source data for Fig. 2d, e.

File name: Supplementary Data 4
Description: Differential expressed gene analysis of RNA-Seq data across all different comparisons (i.e. BD versus PD; BI versus PI; BJQ versus PJQ) and normalized gene expression. BD, bevacizumab + DMSO; PD, palivizumab + DMSO; BI, bevacizumab + I-BET762; PI, palivizumab + I-BET762; BJQ, bevacizumab + JQ1; PJQ, palivizumab + JQ1. All data were from three independent experiments. Source data for Fig. 6b, c.

File name: Supplementary Data 5
Description: Gene set enrichment analysis of RNA-Seq data across all different comparisons (i.e. B_DMSO versus P_DMSO; B_IBET versus P_IBET; B_JQ1 versus P_JQ1). Data represent gene set enrichment in P_DMSO, P_IBET or P_JQ1. All data were from three independent experiments. Source data for Fig. 6d, e.

File name: Supplementary Data 6
Description: Source data for Fig. 1c; Fig. 2b, c; Fig. 3d, f; Fig. 4a–c, e, f; Fig. 5b–g; Fig. 7a, b; Supplementary Figure 3d, e and Supplementary Figure 5b, c.
