# Supplementary material for: Three-dimensional CRISPR screening reveals epigenetic interaction with anti-angiogenic therapy
Source: Commun Biol. 2021 Jul 15;4:878. doi: 10.1038/s42003-021-02397-3 (PMC8282794; doi:10.1038/s42003-021-02397-3)
Supplement: Supplementary file 10 — Reporting Summary [file 42003_2021_2397_MOESM10_ESM.pdf]

nature research

Corresponding author(s): Steven Stacker

Last updated by author(s): Jun 20, 2021

Reporting Summary

Nature Research wishes to improve the reproducibility of the work that we publish. This form provides structure for consistency and transparency in reporting. For further information on Nature Research policies, see our Editorial Policies and the Editorial Policy Checklist.

Statistics

For all statistical analyses, confirm that the following items are present in the figure legend, table legend, main text, or Methods section.

n/a	Confirmed	
☐	☒	The exact sample size (*n*) for each experimental group/condition, given as a discrete number and unit of measurement
☐	☒	A statement on whether measurements were taken from distinct samples or whether the same sample was measured repeatedly
☐	☒	The statistical test(s) used AND whether they are one- or two-sided<br>*Only common tests should be described solely by name; describe more complex techniques in the Methods section.*
☒	☐	A description of all covariates tested
☐	☒	A description of any assumptions or corrections, such as tests of normality and adjustment for multiple comparisons
☐	☒	A full description of the statistical parameters including central tendency (e.g. means) or other basic estimates (e.g. regression coefficient) AND variation (e.g. standard deviation) or associated estimates of uncertainty (e.g. confidence intervals)
☐	☒	For null hypothesis testing, the test statistic (e.g. *F*, *t*, *r*) with confidence intervals, effect sizes, degrees of freedom and *P* value noted<br>*Give P values as exact values whenever suitable.*
☒	☐	For Bayesian analysis, information on the choice of priors and Markov chain Monte Carlo settings
☒	☐	For hierarchical and complex designs, identification of the appropriate level for tests and full reporting of outcomes
☐	☒	Estimates of effect sizes (e.g. Cohen's *d*, Pearson's *r*), indicating how they were calculated

Our web collection on statistics for biologists contains articles on many of the points above.

Software and code

Policy information about availability of computer code

Data collection	No custom code, algorithm or software has been used during this study. Please see Methods and Supplementary Methods for descriptions in detail.
Data analysis	No custom code, algorithm or software has been used during this study. Please see Methods and Supplementary Methods for descriptions in detail.

For manuscripts utilizing custom algorithms or software that are central to the research but not yet described in published literature, software must be made available to editors and reviewers. We strongly encourage code deposition in a community repository (e.g. GitHub). See the Nature Research guidelines for submitting code & software for further information.

Data

Policy information about availability of data

All manuscripts must include a data availability statement. This statement should provide the following information, where applicable:

- Accession codes, unique identifiers, or web links for publicly available datasets
- A list of figures that have associated raw data
- A description of any restrictions on data availability

The RNA-Seq dataset has been deposited in Gene Expression Omnibus (GEO) with the accession number GSE176149 (https://www.ncbi.nlm.nih.gov/geo/query/acc.cgi?acc=GSE176149). Relevant data are available in the article and the Supplementary Information. Source data for the RNA-Seq analysis (Fig. 3a; Fig. 6b–e and Supplementary Figure 2b) are available in Supplementary Data 1, 4 and 5. Source data for the CRISPR screen analysis (Fig. 2d, e) are available in Supplementary Data 2 and 3. Source data for the other figures (Fig. 1c; Fig. 2b, c; Fig. 3d, f; Fig. 4a–c, e, f; Fig. 5b–g; Fig. 7a, b; Supplementary Figure 3d, e and Supplementary Figure 5b, c) are available in Supplementary Data 6. Additional relevant data are available from the corresponding author upon reasonable request.

Field-specific reporting

Please select the one below that is the best fit for your research. If you are not sure, read the appropriate sections before making your selection.

- [x] Life sciences
- [ ] Behavioural & social sciences
- [ ] Ecological, evolutionary & environmental sciences

For a reference copy of the document with all sections, see nature.com/documents/nr-reporting-summary-flat.pdf

Life sciences study design

All studies must disclose on these points even when the disclosure is negative.

Sample size	In our CRISPR screen, we have infected the cells and prepared the samples to ensure an average coverage of at least 2,000 cells per single guide RNA. This is much higher than the numbers that were used in most CRISPR screening studies (e.g. 200–400). And we have four individual single guide RNA targeting each gene in the library we used.
Data exclusions	There were no data exclusions.
Replication	All findings have been validated from multiple independent experiments except that the results in Figure 2c and Supplementary Figure 3d, e were from one experiment.
Randomization	Randomization was not relevant to this study.
Blinding	Blinding was not relevant to this study.

Reporting for specific materials, systems and methods

We require information from authors about some types of materials, experimental systems and methods used in many studies. Here, indicate whether each material, system or method listed is relevant to your study. If you are not sure if a list item applies to your research, read the appropriate section before selecting a response.

Materials & experimental systems

n/a	Involved in the study	
[ ]	[x]	Antibodies
[ ]	[x]	Eukaryotic cell lines
[x]	[ ]	Palaeontology and archaeology
[x]	[ ]	Animals and other organisms
[x]	[ ]	Human research participants
[x]	[ ]	Clinical data
[x]	[ ]	Dual use research of concern

Methods

n/a	Involved in the study	
[x]	[ ]	ChIP-seq
[ ]	[x]	Flow cytometry
[x]	[ ]	MRI-based neuroimaging

Antibodies

Antibodies used	Mouse monoclonal anti-alpha-tubulin (TU-02), Santa Cruz, #sc-8035, 1:200<br>Rabbit monoclonal anti-GAPDH (14C10), Cell Signaling Technology, #2118, 1:2000<br>Mouse monoclonal anti-FLAG M2, Sigma, #F3165, 1:5000
Validation	Please see the citation information on the manufacturer's website.<br><br>Mouse monoclonal anti-alpha-tubulin (TU-02), Santa Cruz, #sc-8035: https://www.scbt.com/p/alpha-tubulin-antibody-tu-02<br>Rabbit monoclonal anti-GAPDH (14C10), Cell Signaling Technology, #2118: https://www.cellsignal.com/products/primary-antibodies/gapdh-14c10-rabbit-mab/2118<br>Mouse monoclonal anti-FLAG M2, Sigma, #F3165: https://www.sigmaaldrich.com/catalog/product/sigma/f3165

Eukaryotic cell lines

Policy information about cell lines

Cell line source(s)	The XSEB113C1 cell line was licensed from Lonza and HEK293T was from Open Biosystems.
Authentication	The XSEB113C1 cell line has been validated by the manufacturer (Lonza) for important phenotypic features of blood endothelial cells.
Mycoplasma contamination	All cell lines have been tested by Victoria Infectious Diseases Reference Laboratory, VIC, Australia and confirmed to be

	negative for mycoplasma contamination.
Commonly misidentified lines (See ICLAC register)	No commonly misidentified cell lines were used in this study.

Flow Cytometry

Plots

Confirm that:

- [x] The axis labels state the marker and fluorochrome used (e.g. CD4-FITC).
- [x] The axis scales are clearly visible. Include numbers along axes only for bottom left plot of group (a 'group' is an analysis of identical markers).
- [x] All plots are contour plots with outliers or pseudocolor plots.
- [x] A numerical value for number of cells or percentage (with statistics) is provided.

Methodology

Sample preparation	Cells were pelleted at 250 × g, room temperature for 5 min and the supernatant was aspirated. The cell pellet was resuspended in diluent [20% v/v flow buffer (1× PBS, 20 mM HEPES (Life Technologies), 0.5% w/v bovine serum albumin (Sigma), 0.5 mM EDTA, pH 7.4 at 23 °C), 80% v/v AccuMax (Sigma), EDTA at a final concentration of 2.5 mM]. Cell suspension was mixed in 96-well plates with the pro-fluorescent stains calcein violet-acetoxymethyl ester (CV-AM; final concentration, 160 nM; Life Technologies) and SYTOX Red (final concentration, 5 nM; Life Technologies) in each well. The plates were incubated at room temperature in the dark for 15 min then analyzed by volumetric flow cytometry.<br><br>Nuclei were released, counted and analyzed as described in He MYC, Stacker SA, Rossi R, Halford MM. Counting nuclei released from microcarrier-based cultures using pro-fluorescent nucleic acid stains and volumetric flow cytometry. BioTechniques 63, 34-36 (2017).<br>Details: after microcarriers in a sample sedimented, the supernatant was removed and the microcarriers were washed with 1× PBS twice. Nuclei were released using cell lysis solution (0.1 M citric acid (Sigma), 1% v/v IGEPAL CA-630 (Sigma)) with vortexing and the microcarriers were then removed by applying the microcarrier slurry to a cell sieve (pore size, 70 μm) followed by a spin at 150 × g, room temperature for 1 min. The nuclei suspension was collected and the volume was measured. If not analyzed immediately, the nuclei suspension was stored at 4 °C for up to two weeks. Nuclei suspension was mixed with the pro-fluorescent nucleic acid stain SYTOX Red (final concentration, 5 nM) in each tube or well. Nuclei counting was performed by volumetric flow cytometry in an experiment format using 5 mL polystyrene tubes or in an assay format using a 96-well plate.
Instrument	FACSVerse flow cytometer, BD Biosciences
Software	Data acquisition was performed using FACSuite software (BD Biosciences) and analysis was performed using FlowLogic software (version 600.0A; Inivai Technologies, Mentone, VIC, Australia) or FlowJo software (version 10.0.8r1; FlowJo, LLC, Ashland, OR, USA).
Cell population abundance	No cell sorting was performed.
Gating strategy	Please see Figure 3c for descriptions in detail.

- [ ] Tick this box to confirm that a figure exemplifying the gating strategy is provided in the Supplementary Information.
